# Dundee Royal Infirmary

**Published:** 1888-12-01

**Authors:** R. Neaves M'Cosh

**Affiliations:** the Medical Superintendent


### Dundee Royal Infirmary.

By R. Neaves M'Cosh, M.D., the Medical Superintendent.

In the November 17th number of The Hospital you quote some statistics from the last annual report of the Dundee Royal Infirmary quite correctly. You then, to give a general idea of the Infirmary, apply some epithets to the wards so grotesquely absurd as to be intelligible only on the supposition that your informant is confounding Dundee with some other place. Certainly, no person answering to your description of your critic has within any period that could decently be called recent introduced himself to me. If there is anything that distinguishes Dundee Infirmary among its fellow hospitals, it is its spacious, handsome, and attractive wards. "How bright your wards are" is one of the commonest remarks of visitors and new comers. Would anyone ever dream from your remarkable reference that Dundee Infirmary is a large, handsome structure, standing on rising ground in acres of open space, and with a frontage over a hundred yards long, facing the south, so that its wards must catch every breath of air, and every gleam of sunshine going. Its wards are large, airy, well-lighted, well-warmed, tastefully-coloured, well-furnished, inviting. The children's ward, in particular, is a perfect gem. The bedding and ward linen are excellent, and in abundance. The food is of good quality, and every one in the infirmary gets as much as he or she can eat. Every one in the infirmary, patient, servant, nurse, official is comfortably housed. In no hospital, I fearlessly assert, is the comfort of the patients better seen to; and the Dundee nursing is well known throughout Scotland. You very happily describe your critic's experience as "unique." So is a man's who finds two and two make five. He thinks one thing, and all the rest of the world think another. His experience of Dundee Infirmary is so unique, as to be in direct opposition to the expressed verdict of every medical man who has ever visited the place with me, and also to that of deputations from other hospitals, who have several times subjected it to a searching examination. At the end of each such visit, it is quite usual to hear from the observer, "Well, there is no want of money about this Infirmary. The Dundee people must be good to you." I am proud now to be able to add to the usual acknowledgment that last year the working people alone gave us very nearly £2,000—a curious commentary on the "repellent" character of the house. The only explanation that can ever be imagined for your critic's blundering, if he refers to Dundee at all, is this: From an architectural defect in the original building, which has caused the present architect and the directors many an hour's trouble trying to find a remedy for, the entrance-hall of the Infirmary is rather dark and dingy, and it is just possible that a very careless visitor, but only a very careless visitor, might import his recollection of the hall into his recollection of the wards, with which it has nothing to do. You critic may take what comfort he can out of my considerate help. Your own reference to our directors is quite in keeping with your critic's reference to our wards. Our directors are a body of shrewd, practical, business men, who devote quite an exceptional amount of time and care to the interests of their Infirmary. That they are not slow to see defects, and that they are ready to spend money freely to remedy them, is evident from this: that in the last ten years £14,000 has been spent in improving the hospital—what is called extraordinary expenditure—and in the last three years, £3,500. But they will certainly not throw away £8 per bed, even for the comical reason you give—to encourage others to do the same. It seems to be a sore point with some people that in Dundee we can run a good infirmary—well, for less money than some of our neighbours can; but I am in hope that before I leave Dundee this long-talked-of Hospital Inquiry Commission may be at work, and then we shall hear something about this £8 per bed that will be startling to London ears. It seems a pity that a magazine, whose very existence depends on its accuracy, should, without any previous attempt at verification, lightly permit itself such ill-considered statements.

